# Prognostic Significance of *HMGA1* Expression in Lung Cancer Based on Bioinformatics Analysis

**DOI:** 10.3390/ijms23136933

**Published:** 2022-06-22

**Authors:** Lias Saed, Agnieszka Jeleń, Marek Mirowski, Aleksandra Sałagacka-Kubiak

**Affiliations:** Department of Pharmaceutical Biochemistry and Molecular Diagnostics, Medical University of Lodz, Muszyńskiego 1, 90-151 Lodz, Poland; lias.saed@umed.lodz.pl (L.S.); agnieszka.jelen@umed.lodz.pl (A.J.); marek.mirowski@umed.lodz.pl (M.M.)

**Keywords:** high-mobility group protein, methylation, prognosis, survival, protein–protein interaction network

## Abstract

High-mobility group protein 1 (HMGA1) participates in the processes of DNA transcription, replication, recombination, and repair. The *HMGA1* gene is expressed abundantly during embryogenesis and is reactivated during carcinogenesis. *HMGA1* gene expression has been associated with a high degree of malignancy, metastatic tendency, and poor survival in breast, colon, ovary, and pancreatic cancers. However, its prognostic significance in lung cancer remains unclear. Using publicly available data, *HMGA1* was shown to be overexpressed in both small and non-small lung tumors, with higher expression compared to both the adjacent non-malignant lung tissues and non-tumor lung tissues of healthy individuals. Elevated *HMGA1* expression could result from lowered *HMGA1* methylation and was connected with some clinicopathological features like sex, age, and stage of the disease. The high *HMGA1* expression level was connected with shorter overall and first progression survival time among lung adenocarcinoma patients, but not lung squamous cell carcinoma patients. HMGA1 could interact with proteins involved in cellular senescence and cell cycle control (TP53, RB1, RPS6KB1, and CDK1), transcription regulation (EP400 and HMGA2), chromatin assembly and remodeling (LMNB1), and cholesterol and isoprene biosynthesis (HMGCR and INSIG1). Taken together, *HMGA1* overexpression could be an essential element of lung carcinogenesis and a prognostic feature in lung cancer.

## 1. Introduction

Despite recent advances in the understanding of the molecular pathogenesis and treatment of lung cancer, it remains one of the most commonly diagnosed and most deadly neoplasms worldwide [1]. Among lung cancer patients, survival is strongly influenced by the clinical stage. Unfortunately, due to the lack of efficient diagnostic methods, early detection of cancer, when currently available treatment schemes are most effective, is rare. The development of lung cancer is a multi-annual and multi-stage process that could be initiated by several carcinogens, as well as by hormonal and genetic features [2,3]. However, too little is still known about host factors facilitating the initiation and progression of tumor development and determining the individual response to treatment. Thus, the search for new, more effective, and precise molecular diagnostic and prognostic markers, as well as drug targets, for lung cancer is an urgent need. 

High-mobility group protein 1 (HMGA1) is a non-histone chromosomal protein involved in the processes of DNA transcription, replication, recombination, and repair. As the HMGA1 protein regulates gene expression and alters the structure of chromatin, it has been described as an architectural transcription factor. It possesses three independent domains (AT-hook) in its structure; these bind to the minor groove of the DNA double helix in the regions of promoters and sequences enhancing the transcription of genes rich in AT sequences [4,5]. The *HMGA1* gene is expressed abundantly during embryogenesis, but at low or even undetectable levels in mature differentiated tissues [6]. The gene is also reactivated during the process of cancer formation. *HMGA1* overexpression has been noted in in breast, colon, ovarian, and pancreatic cancer, among others [7]. For many malignant neoplasms, the *HMGA1* gene has been associated with a high degree of malignancy, resistance to chemotherapy, metastatic tendency, and low patient survival [8]. Studies also show that HMGA1 has oncogenic properties in cultured cells and transgenic mice, but how the protein leads to neoplastic transformation is not fully elucidated [8,9].

The data about the role of the *HMGA1* gene and its protein in lung cancer pathogenesis and prognostication is also limited. Expression of both the *HMGA1* gene and the HMGA1 protein has been found to be upregulated in various lung cancer cell lines in comparison with normal human lung bronchial epithelium [10,11]. Correspondingly, higher expression of the *HMGA1* gene has been noted in most primary lung tumors compared to normal lung tissue [10,11,12]. HMGA1 protein was found in an increased amount in metaplasia, dysplasia, and carcinoma in situ of the bronchial epithelium [13]. High HMGA1 protein expression in NSCLC tissue was connected with a higher TNM stage [14] and *HMGA1* gene overexpression with shorter overall survival [11] and increased HMGA1 protein levels [15] in tumor tissue among NSCLC patients. However, some contradictory findings were also published [16]. Taken together, it appears that *HMGA1* can play an important role in pulmonary carcinogenesis and also could be a promising prognostic biomarker or therapeutic target in lung cancer. However, the above-mentioned studies were conducted in relatively small sample groups with different methodologies; as such, it is not possible to draw clear conclusions about the significance of *HMGA1* for lung cancer. 

Therefore, the present study attempts to integrate data about *HMAG1* gene expression in lung cancer from several publicly available databases. A variety of bioinformatical analyses were performed, including screening for *HMGA1* expression levels across different malignant tumors, comparing *HMGA1* expression between lung cancer and normal pulmonary tissue, evaluating the association between the level and lung cancer clinical features and prognosis, and constructing a functional network of the HMGA1 protein. 

## 2. Results

### 2.1. An Increase in HMGA1 mRNA Expression Level and HMGA1 Protein Amount Is Common in Multiple Carcinomas

**Figure 1:**
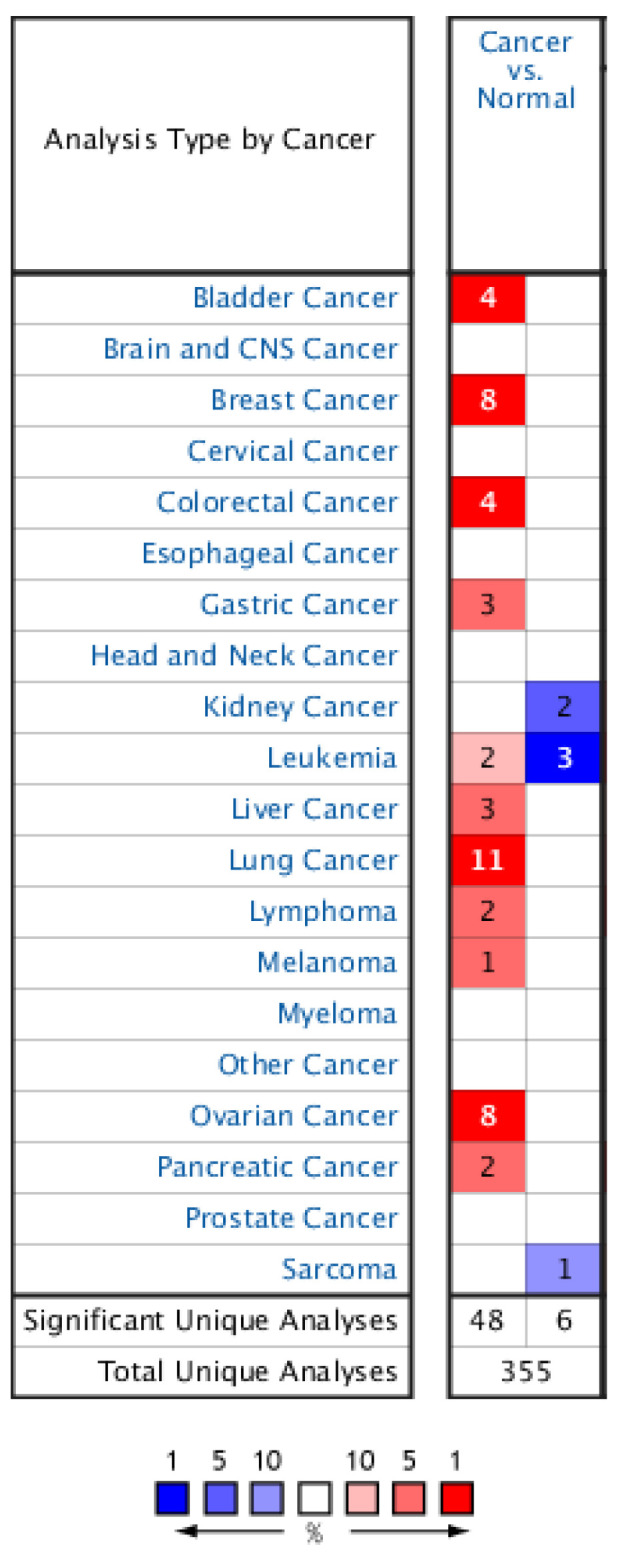
Differential mRNA level of *HMGA1* in 20 types of cancer compared to matched normal tissue in the Oncomine. Significantly (*p* < 0.05) increased and decreased levels of *HMGA1* are indicated in red and blue, respectively. The intensity of cell color is determined by the best gene rank percentile for the analyses within the cell. The number in each cell represents the number of analyses that meet the given thresholds within the analysis and cancer types.

**Figure 2:**
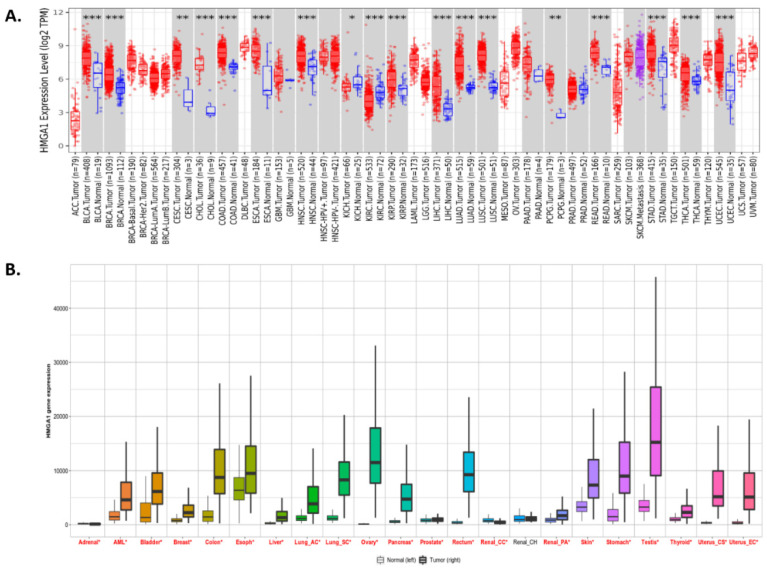
*HMGA1* expression levels in different cancer types compared to the corresponding normal tissue: (**A**) TIMER2.0; the statistical significance (*p*-value) computed by the Wilcoxon test is annotated by the number of stars: * *p* < 0.05; ** *p* < 0.01; *** *p* < 0.001; box plots in grey columns indicate cancer types where data for matched normal tissue was available; red and blue box plots indicate tumor and normal samples, respectively (**B**) TNMplot; cancer names where differences with *p* < 0.01 was detected are typed in red.

**Figure 3:**
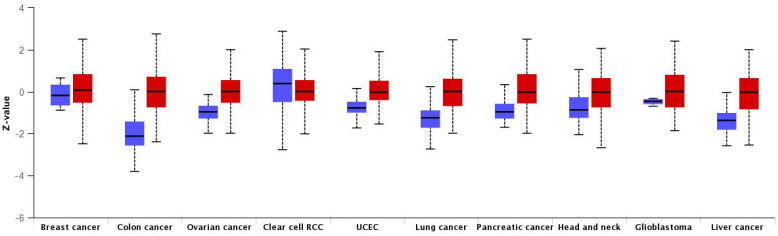
The HMGA1 protein levels in different cancer types compared to normal tissues investigated by UALCAN. Blue and red box plots indicate normal and tumor samples, respectively.

First, the differential expression of the *HMGA1* between various cancers and noncancerous tissues from healthy individuals was assessed via the Oncomine platform (Figure 1). As shown in Figure 1, increased mRNA expression (red) was observed in most of the analyzed cancers, e.g., in bladder, breast, ovarian, and gastrointestinal tumors. Eleven of 34 analyses in 9 of 14 datasets collected in Oncomine found *HMGA1* to belong to 10% of the top overexpressed genes in lung cancer. A similar observation was made using the TIMER2.0 database (Figure 2A) where lung cancer was found to be among the neoplasms most significantly overexpressing the *HMGA1* (*p* < 0.001). Analysis of RNA-seq data available via the TMNplot web tool produced similar results to those described above (Figure 2B). These elevated mRNA expression levels were associated with an increase in HMGA1 protein levels compared to non-cancerous tissue in the investigated cancer types (Figure 3). One exception was renal clear cell carcinoma, where a decrease in HMGA1 protein was accompanied by a decline in *HMGA1* mRNA expression. 

### 2.2. HMGA1 Is Overexpressed in Lung Cancer in Comparison to Both Adjacent and Unpaired Normal Lung Tissue

The TNMplot platform tool was used to compare the *HMGA1* expression level between paired lung cancer tumors and adjacent normal tissue collected from the same patients and between non-paired tumors and normal lung tissues collected from separate subject cohorts. Statistically significant overexpression of the studied gene was noted in the cancerous tissue in comparison to both adjacent and unpaired lung tissue (*p* = 1.36 × 10 ^−29^ and *p* = 8.66 × 10^−54^, respectively). Significantly higher *HMGA1* expression was also stated in both lung adenocarcinoma and squamous cell carcinoma compared to adjacent samples (*p* = 5.19 × 10^−10^ and *p* = 1.66 × 10^−9^, respectively) and unpaired samples (*p* = 3.59 × 10^−96^ and *p* = 1.36 × 10^−152^, respectively).

**Figure 4:**
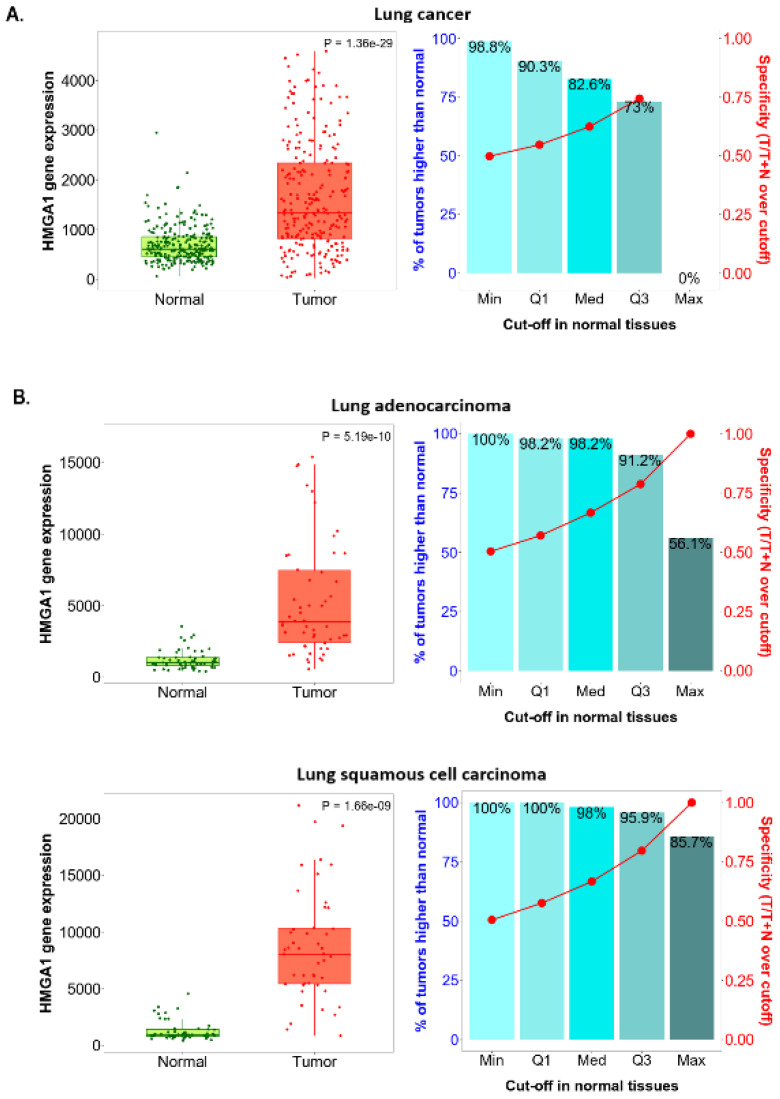
*HMGA1* expression level in paired tumor and adjacent normal tissue by TNMplot. (**A**) Gene chip data for lung cancer. (**B**) RNA-Seq data for adenocarcinoma and squamous cell carcinoma.

**Figure 5:**
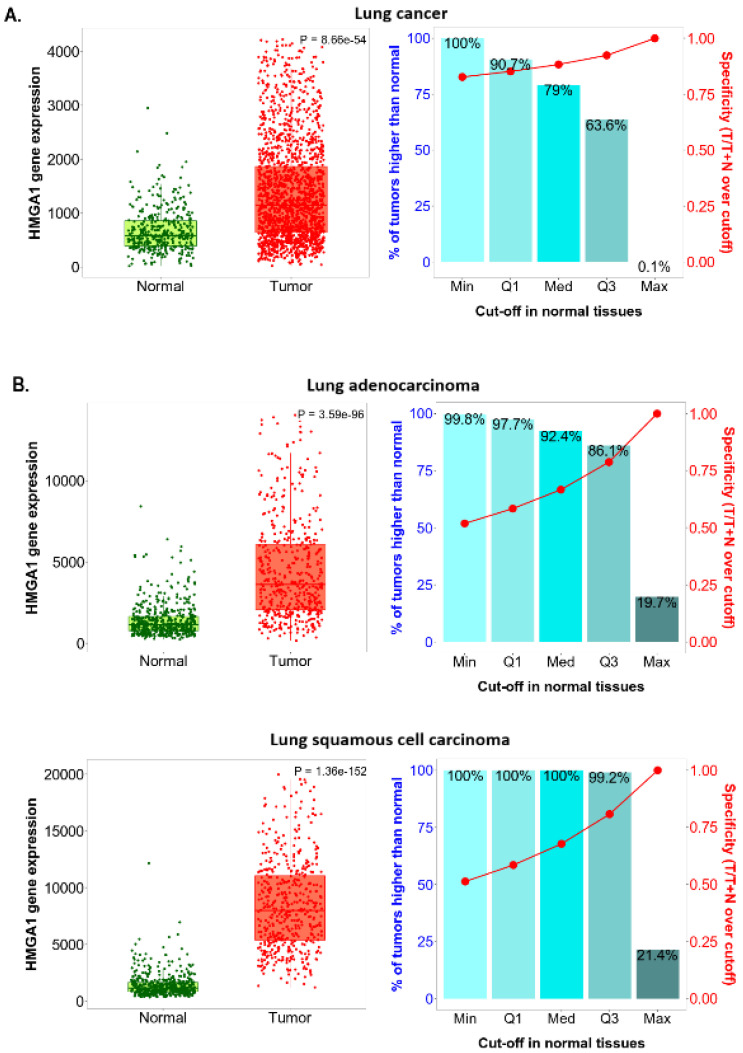
*HMGA1* expression level in non-paired tumor and normal tissue by TNMplot. (**A**) Gene chip data for lung cancer. (**B**) RNA-Seq data for adenocarcinoma and squamous cell carcinoma.

The right-sided bar charts in Figure 4 and Figure 5 also provide a graphical representation of the sensitivity and specificity of *HMGA1* expression level at the major cut-offs set on the base of the range of the *HMGA1* expression level in normal samples. In lung cancer samples, a combination of the highest sensitivity and specificity was found at the third quartile cut-off when referencing adjacent normal tissue (Figure 4A) and at the median cut-off when referencing unpaired normal tissue (Figure 5A). When the lung adenocarcinoma and squamous cell carcinoma subtypes are analyzed individually, even better sensitivity and specificity values were calculated at the third quartile cut-off, referencing both adjacent and unpaired normal tissues (Figure 4B and Figure 5B). 

### 2.3. HMGA1 Is Widely Overexpressed in Different Histological Types of Lung Tumour

**Figure 6:**
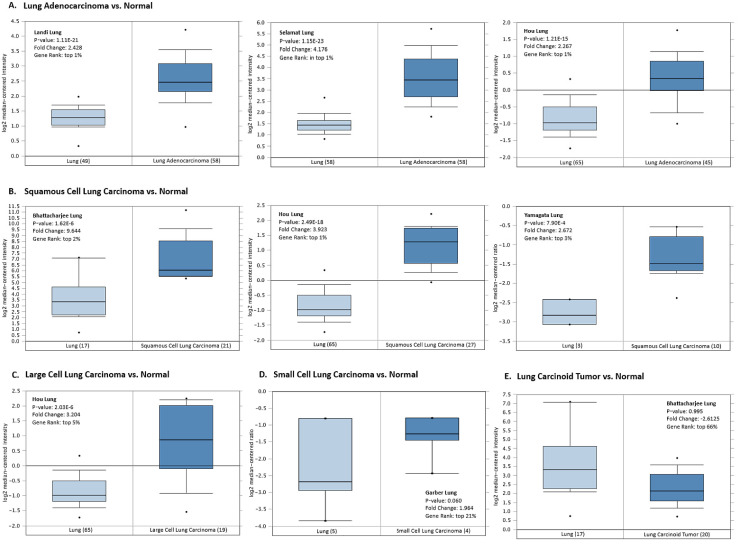
*HMGA1* expression level in normal lung tissue and different histological subtypes of lung neoplasm by Oncomine platform. The analysis is shown for (**A**) lung adenocarcinoma (Landi Lung, Selemat Lung, Hou Lung), (**B**) squamous cell lung carcinoma (Bhattecharjee Lung, Hou Lung, Yamagata Lung), (**C**) large cell lung carcinoma (Hou Lung), (**D**) small cell lung carcinoma (Garber Lung), and (**E**) lung carcinoid tumor (Yamagata Lung).

The histological characteristics of the lung cancer tissue substantially influence the behavior of neoplastic cells, determine cancer prognosis, and direct treatment regimens applied. Thus, we explored the *HMGA1* mRNA expression data from Oncomine related to small cell lung cancer, the main three subtypes of non-small cell lung cancer, and lung carcinoids. Consistent *HMAG1* overexpression was found across different non-small cell lung cancer subtypes: adenocarcinoma (Figure 6A), squamous cell carcinoma (Figure 6B), and large cell carcinoma (Figure 6C). *HMGA1* upregulation was observed in small cell carcinoma, but without statistical significance (Figure 6D). Contrarily, no significant difference in *HMGA1* expression level was detected between lung carcinoid tumors and non-cancerous lung tissue (Figure 6E). 

**Figure 7:**
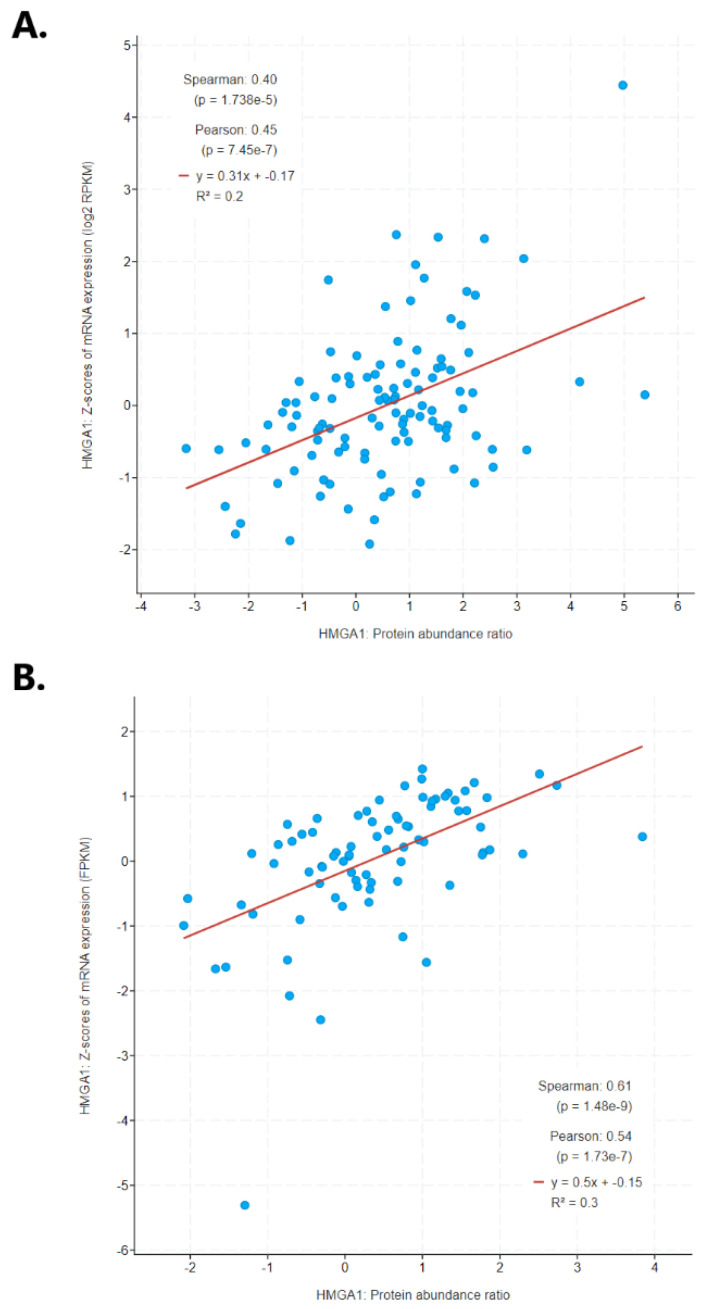
Correlation between the *HMGA1* mRNA expression level and HMGA1 amount ratio in (**A**) lung adenocarcinoma and (**B**) lung squamous cell carcinoma, calculated using cBioPortal.

The study also examined whether an increase in *HMGA1* mRNA expression in lung cancer is followed by an elevation of HMGA1 protein. Indeed, statistically significant correlations found between mRNA expression score and protein abundance for lung cancer data collected by cBioPortal. An increase in *HMGA1* mRNA level was associated with a proportional gain in the HMGA1 protein amount in both lung adenocarcinoma (R Spearman 0.40, *p* < 0.001; R Pearson 0.45 *p* < 0.001; Figure 7A) and lung squamous cell carcinoma (R Spearman 0.61, *p* < 0.001; R Pearson 0.54, *p* < 0.001; Figure 7B).

### 2.4. HMGA1 Expression Level in Lung Cancer Could Be Connected with Methylation Status but Not with DNA Alterations of the HMGA1 Gene

**Figure 8:**
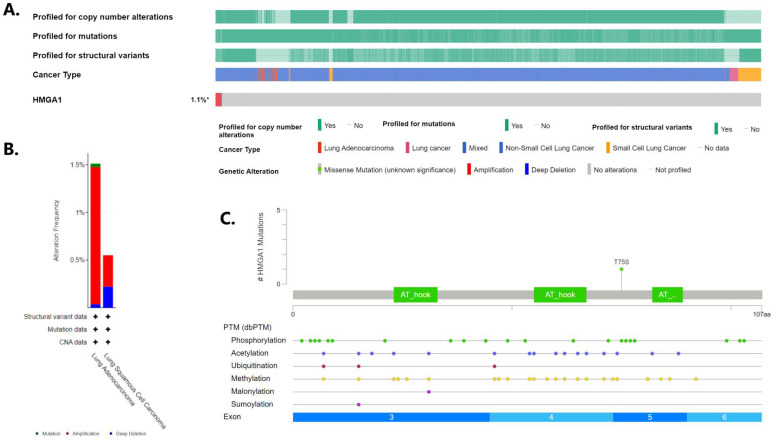
Genomic alterations of *HMGA1* in lung cancer according cBioPortal. (**A**) Oncoprint of the *HMAG1* in lung cancer, (**B**) Incidence of different alterations according to NSCLC types, (**C**) Details of *HMGA1* missense mutation found in lung adenocarcinoma.

**Figure 9:**
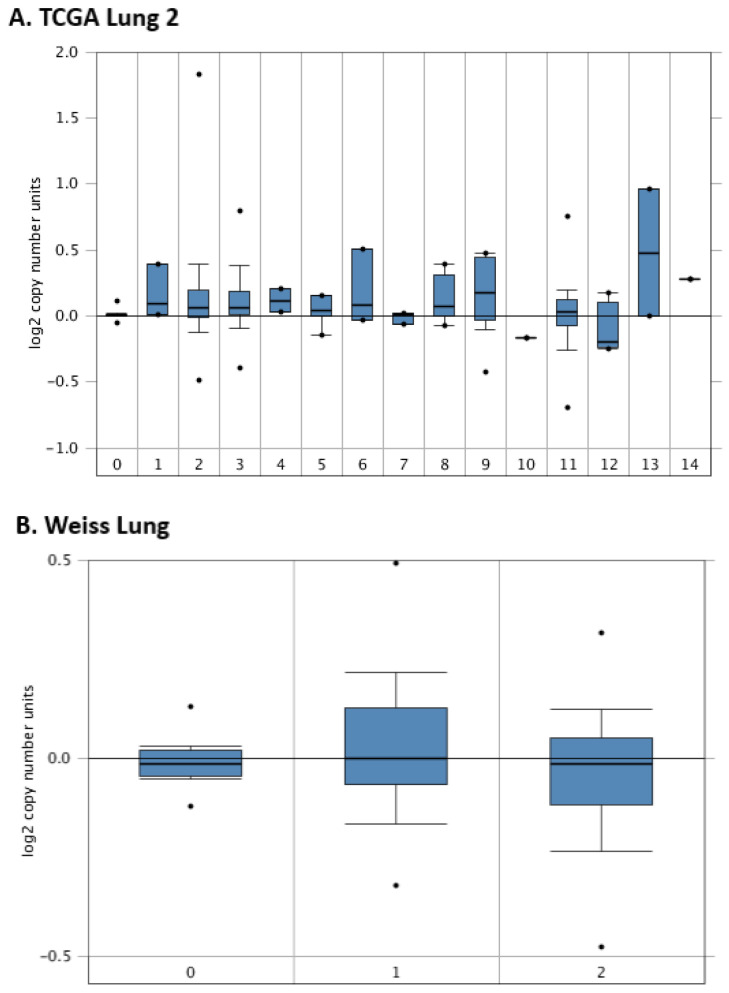
*HMGA1* copy number in normal lung tissue and different histological subtypes of lung neoplasm by Oncomine: (**A**) TCGA lung 2: 0. normal lung (*n* = 810), 1. acinar lung (*n* = 6), 2. lung adenocarcinoma (*n* = 261), 3. lung adenocarcinoma, mixed subtype (*n* = 67), 4. lung clear cell adenocarcinoma (*n* = 2), 5. lung mucinous adenocarcinoma (*n* = 6), 6. micropapillary lung adenocarcinoma (*n* = 3), 7. mucinous bronchioloalveolar carcinoma (*n* = 3), 8. non-mucinous bronchioloalveolar carcinoma (*n* = 9), 9. papillary lung adenocarcinoma (*n* = 10), 10. solid lung adenocarcinoma (*n* = 1), 11. squamous cell lung carcinoma (*n* = 348), 12. squamous cell lung carcinoma, basaloid variant (*n* = 8), 13. squamous cell lung carcinoma, papillary variant (*n* = 2), 14. squamous cell lung carcinoma, small cell variant (*n* = 1); (**B**) Weiss lung: 0. normal lung (*n* = 59), 1. lung adenocarcinoma (*n* = 77), 2. squamous cell lung carcinoma (*n* = 155).

**Figure 10:**
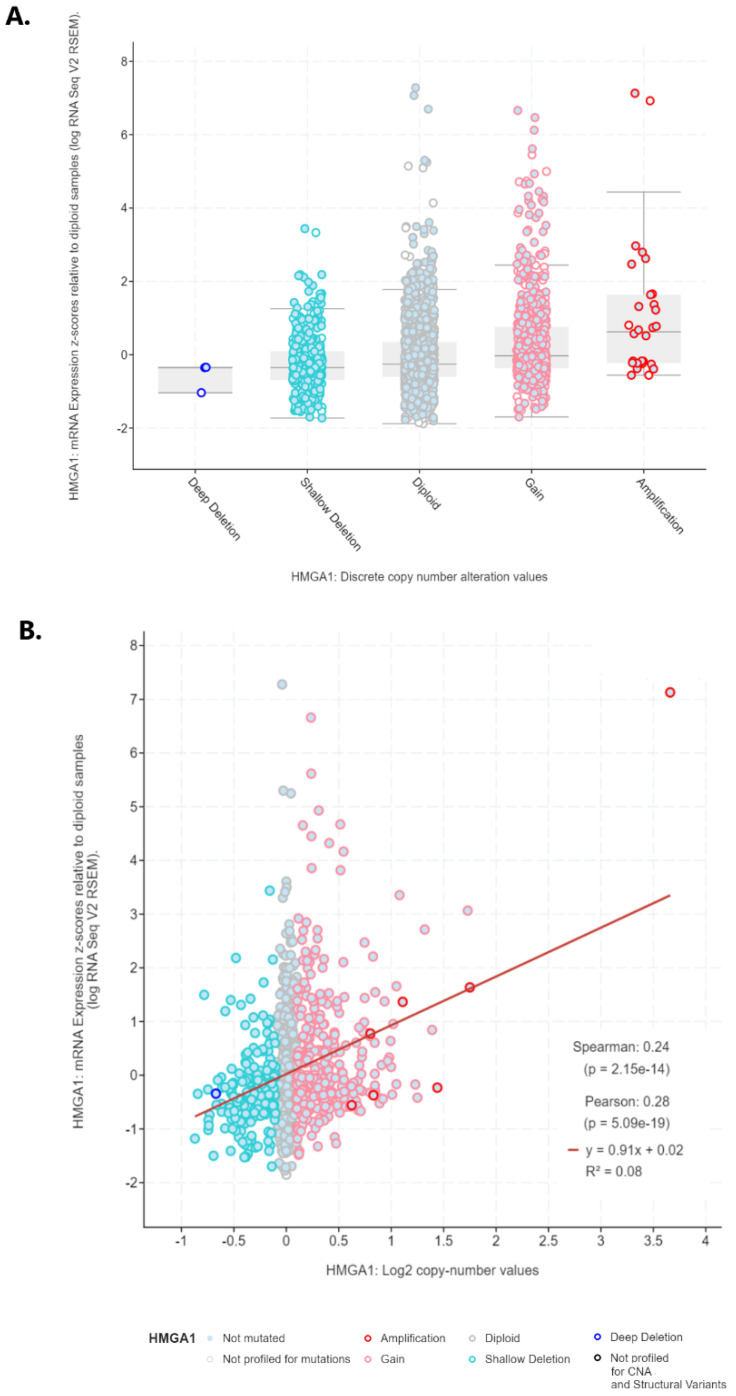
Association between *HMGA1* mRNA expression level and (**A**) discrete copy number alterations, (**B**) log2 copy-number value (generated by cBioPortal).

*HMGA1* overexpression observed at the mRNA level is a common feature of lung cancer. One of the reasons for this may be changes in the sequence or the copy number of the *HMGA1* gene. Therefore, Oncoprint was generated by using cBioPortal to query 7276 patients/7799 samples from 27 studies (Figure 8A). It revealed that *HMGA1* gene alterations were stated in only 1.1% of lung cancer patients profiled for copy number changes, mutations, and structural variants of the gene, namely in about 1.5% of lung adenocarcinoma and 0.5% of lung squamous cell carcinoma cases. Among them, the most frequently detected were gene amplification, with deep deletions less frequently (Figure 8B). The only mutation reported was the T75S missense mutation in lung adenocarcinoma (Figure 8C). The data from Oncomine confirmed no substantial change in copy number variation of *HMGA1* across different lung cancer subtypes (Figure 9). However, plotting *HMGA1* expression level against copy number alterations clearly showed an increase in the level from deep deletion to amplification (Figure 10A). A statistically significant positive correlation was noted between *HMGA1* expression level and log2 copy-number value (R Spearman 0.24, *p* < 0.001; R Pearson 0.28, *p* < 0.001; Figure 10B).

**Figure 11:**
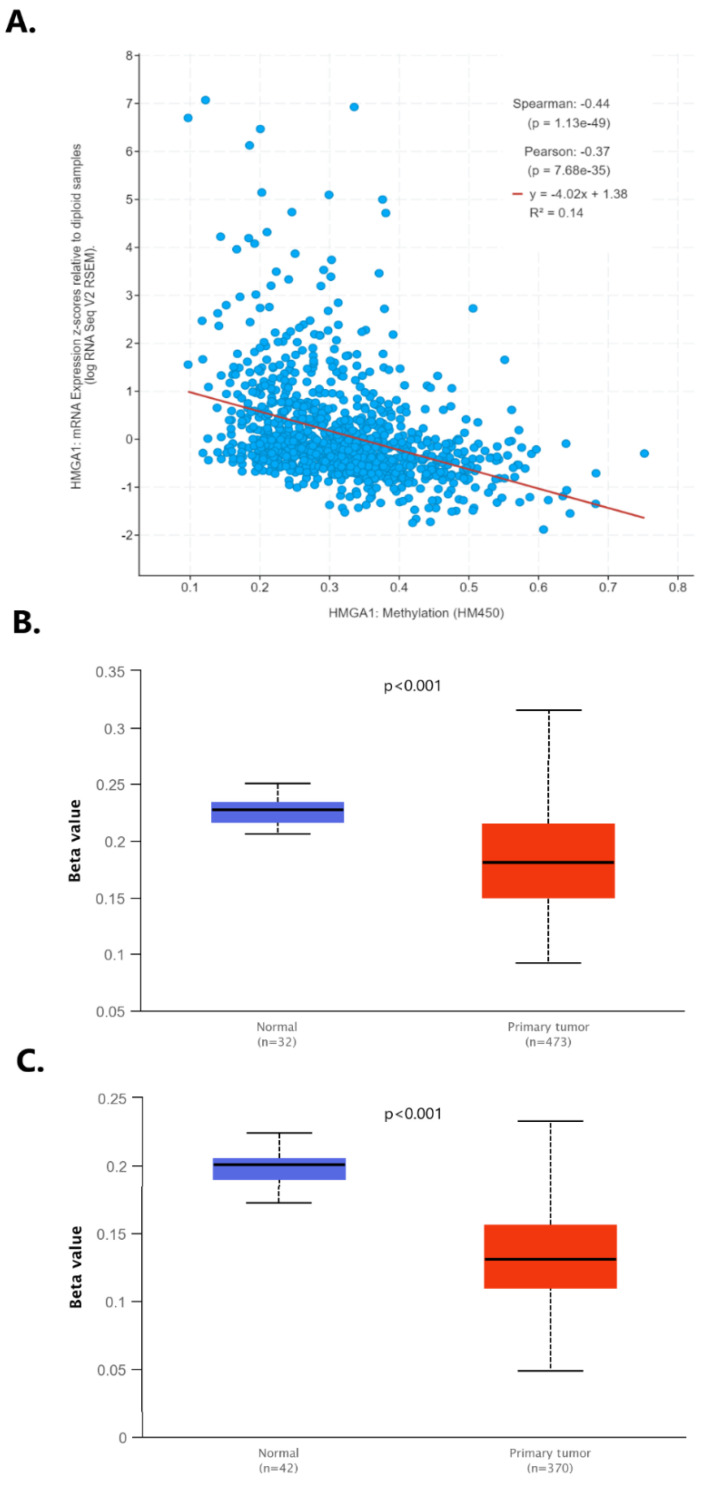
(**A**) Correlation between the mRNA expression level and DNA methylation profile of the *HMGA1* in lung cancer by cBioPortal; the promoter methylation level of the *HMGA1* in (**B**) lung adenocarcinoma and (**C**) lung squamous cell carcinoma tissue in comparison with normal lung tissue by UALCAN.

As mutations or copy number changes of the *HMGA1* appear to occur rarely in lung cancer, potential causes of *HMGA1* overexpression were sought. Since DNA methylation can change the gene activity without changing the sequence, the connection between the *HMGA1* expression level and DNA methylation profile in lung cancer was inspected using cBioPortal. The significant negative correlation between the mentioned features was stated (R Spearman −0.44, *p* < 0.001; R Pearson −0.37, *p* < 0.001; Figure 11A). The results regarding *HMGA1* promoter methylation yielded by the UALCAN database were consistent with this association. For both the lung adenocarcinoma and lung squamous cell carcinoma promoter, the methylation level was substantially lowered in the tumor tissue in comparison to normal lung tissue (*p* < 0.001 for both cancer subtypes, Figure 11B). 

### 2.5. HMGA1 Expression Level Is Connected with Selected Clinical Parameters in Lung Cancer

**Figure 12:**
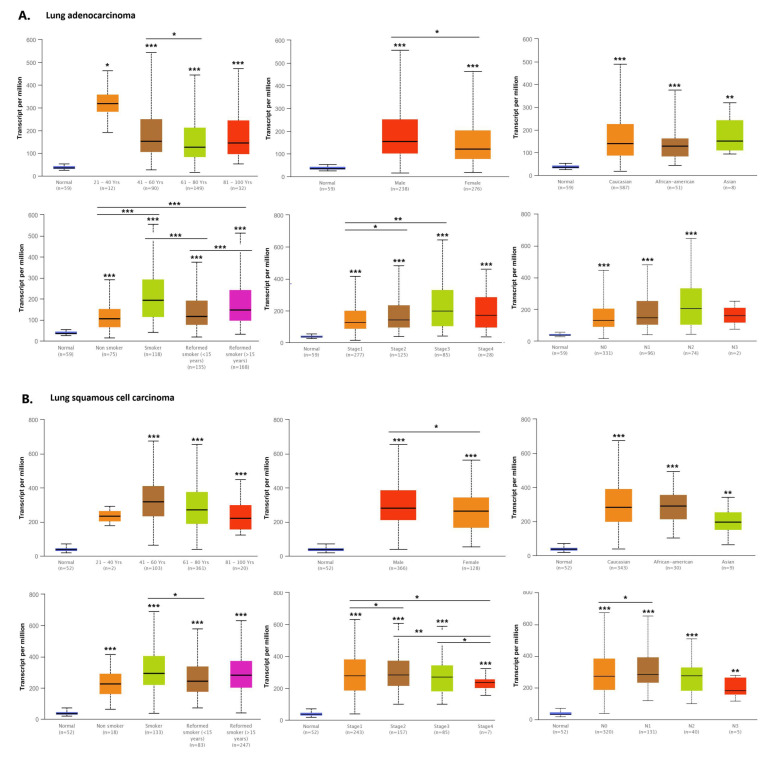
Association between the *HMGA1* expression level and selected clinical parameters: age, gender, population affinity, smoking status, TNM stage, and nodal involvement in healthy individuals and lung cancer patients. (**A**) lung adenocarcinoma patients. (**B**) lung squamous cell carcinoma patients (UALCAN); * *p* < 0.05, ** *p* < 0.01, *** *p* < 0.001; the first layer-* right above the error bar indicates comparison to the normal group, the above layers-* placed above a second line indicates the comparison between corresponding groups covered by the line.

Many factors, including the extent of the spread of neoplastic cells in the body or their histological differentiation, affect the outcome of lung cancer patients. Therefore, to evaluate the clinical significance of the *HMGA1* expression level in lung cancer, *HMGA1* expression level was compared with selected clinicopathological features: age, sex, population affinity, smoking status, TNM stage, and nodal involvement separately in lung adenocarcinoma (Figure 12A) and squamous cell carcinoma (Figure 12B) with the usage of the UALCAN database.

In lung adenocarcinoma, a significantly higher expression level was found in lung adenocarcinoma patient groups aged between 41 and 60 years than 61–80 years (*p* = 0.0335). No differences were found between any two of the remaining age groups. The *HMGA1* expression level was not connected with the age of lung squamous cell carcinoma patients. The higher expression level of the gene was consistently higher in men than women in both lung adenocarcinoma (*p* = 0.0393) and lung squamous cell carcinoma (*p* = 0.0177). Population affinity did not give rise to any significant differences in the *HMGA1* expression in any of the analyzed subtypes of lung cancer.

In both lung adenocarcinoma and squamous cell carcinoma, the level of *HMGA1* expression was the lowest in non-smokers, of intermediate value in reformed smokers, and the highest in current smokers. In lung adenocarcinoma, statistically significant differences were stated between the non-smoker group and any of the groups of reformed smokers >15 years (*p* = 0.0004) and current smokers (*p* = 0.0000). There was also a difference in the level between reformed smokers for <15 years and any of the groups of reformed smokers for >15 years (*p* = 0.0006) and smokers (*p* = 0.0000). In squamous cell carcinoma, only smokers and reformed smokers for <15 year groups differed significantly in *HMGA1* expression level (*p* = 0.0217).

In lung adenocarcinoma, nodal status was not connected with the expression level of the HMGA1. In squamous cell carcinoma, higher expression was noted in the N1 group than in the N0 group (*p* = 0.0232); however, no significant differences were found between other subgroups of patients. In both analyzed subtypes of lung cancer, some dissimilarities in *HMGA1* expression level were stated. In lung adenocarcinoma, the level was significantly lower in stage I than in stages II (*p* = 0.0227) and III (*p* = 0.0023). In squamous cell carcinoma, the *HMGA1* expression level was lower in stage I than in stage II (*p* = 0.0330). Additionally, in this subtype of lung cancer, the expression level in stage IV was significantly lower than the level in the other three subgroups of different clinical stages: stage I (*p* = 0.0314), stage II (*p* = 0.0314), and stage III (*p* = 0.0188).

**Figure 13:**
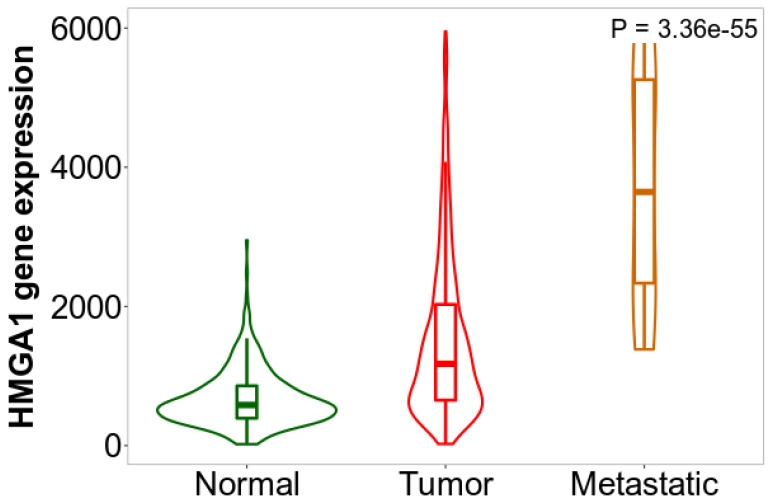
The *HMGA1* expression level in normal lung tissue and lung cancer tissue—primary and metastatic samples. Plot generated using TNMplot based on gene chip data.

Additionally, the gene chip data provided by TNMplot indicated significant differences in *HMGA1* expression level between normal lung tissue and lung cancer tissue-primary and metastatic samples (*p* = 0.0000; Figure 13). The level was significantly lower in normal tissue than in tissues of both primary tumors (*p* = 0.0000) and metastases (*p* = 0.0000). It was also lower in primary tumors than in metastases (*p* = 0.0000).

### 2.6. High HMGA1 Expression Is Related to a Poor Prognosis in Lung Cancer

To evaluate the association between the *HMGA1* expression level and the prognosis of lung cancer patients, a survival analysis was done using the Kaplan–Meier plotter. Lung cancer patients with high expression of the gene had a shorter overall (*p* < 1 × 10^−16^) and first progression (*p* = 1.7 × 10^−6^) survival time in comparison to the low expression group. As an association was found between the *HMGA1* expression level and some clinicopathological factors known to be determinants of patient survival, a survival analysis was performed restricted to subgroups of patients who differed with regard to sex, smoking history, grade, stage, and histological type (Table 1). 

High *HMGA1* expression level was connected with poor overall and first progression survival in both sexes and both analyzed smoking history states. The association between *HMGA1* expression and overall and first progression survival was significant only in stage I but not in more advanced stages. Correspondingly, this connection was stated as significant in AJCC stage T 1 for overall and first progression survival, and AJCC stage T 2 for overall survival, but not in more advanced AJCC stages T. A high *HMGA1* expression level was associated with poor overall survival in AJCC stages N 0 and N1 and with first progression survival in AJCC stages N1 and N2. In AJCC stages M0, the high expression level of the gene was connected with shorter overall, but not first progression, survival.

**Figure 14:**
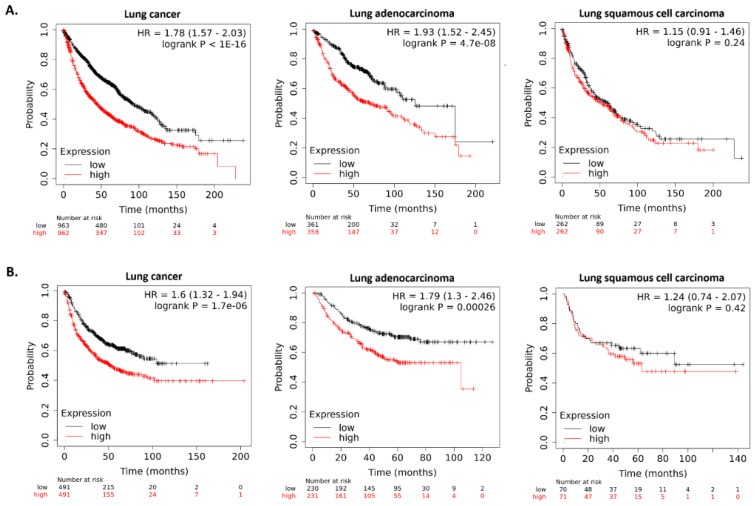
Kaplan–Meier survival curves based on *HMGA1* expression level by Kaplan–Meier plotter: (**A**) the overall survival for all lung cancer patients (*n* = 1925), lung adenocarcinoma patients (*n* = 719), and squamous cell lung carcinoma patients (*n* = 524); (**B**) the first progression survival for all lung cancer patients (*n* = 982), lung adenocarcinoma patients (*n* = 461), and squamous cell lung carcinoma patients (*n* = 141).

A significant connection was observed between the *HMGA1* expression level and overall survival times in lung adenocarcinoma (*p* = 4.7 × 10^−8^), but not in the lung squamous cell carcinoma subtype (*p* = 0.2403; Figure 14A). The first progression survival analysis showed similar results (adenocarcinoma *p* = 0.0003, squamous cell carcinoma *p* = 0.4168, Figure 14B). To validate these findings, the survival data of NSCLC, lung adenocarcinoma, and lung squamous cell carcinoma patients provided by the PrognoScan database was compared (Table 2). For adenocarcinoma, a connection between *HMGA1* expression level and overall survival was detected with Cox *p*-value < 0.1 in nine of fourteen different datasets, and between *HMGA1* expression and relapse-free survival in one of two datasets. For squamous cell carcinoma, no association was found between *HMGA1* expression and neither overall nor relapse-free survival, based on the available six datasets. 

### 2.7. HMGA1 Protein Interacts with Proteins Involved in Cellular Senescence and Ageing, Cell Cycle Control, Transcription Regulation, Chromatin Assembly/Remodelling, and Cholesterol and Isoprene Biosynthesis

**Figure 15:**
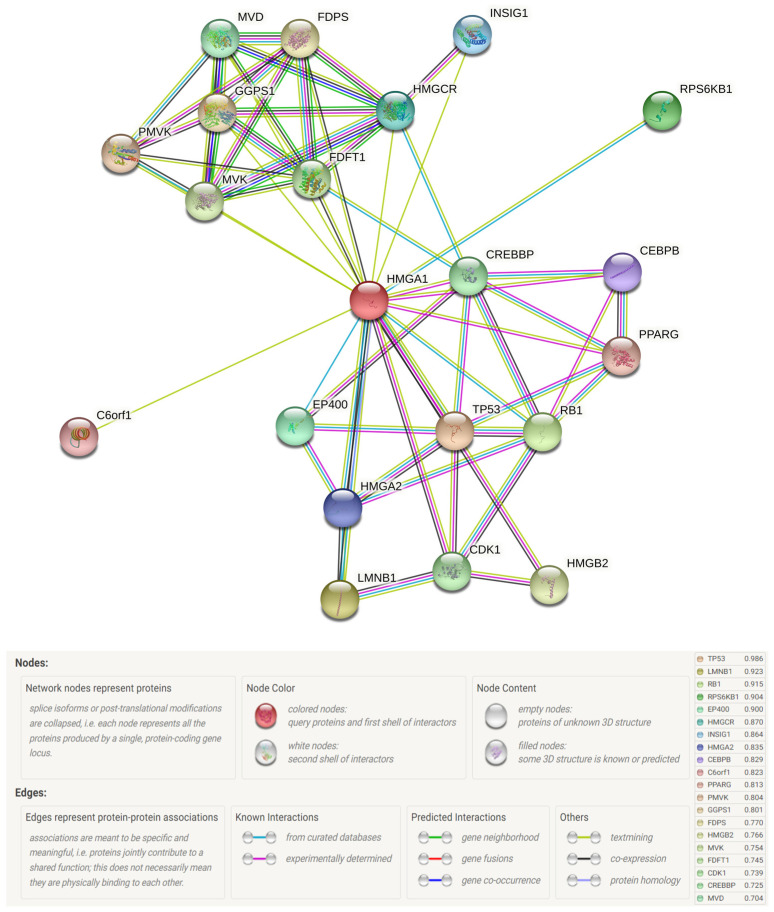
The protein interaction network of the HMGA1 gained from the STRING database.

Lastly, to understand the importance of HMGA1 for the carcinogen process, the study examined the proteins that interact with HMGA1 and the related cellular processes. An HMGA1-centered protein–protein network was constructed using the STRING online tool. Interactors predicted with a confidence score of at least 0.700 are presented in Figure 15. The top functional partners of the HMAG1 protein, predicted with the highest confidence, were cellular tumor antigen p53 (TP53, score = 0.986), lamin-B1 (LMNB1, score = 0.923), retinoblastoma-associated protein (RB1, score = 0.915), ribosomal protein S6 kinase beta-1 (RPS6KB1, score = 0.904), and E1A-binding protein p400 (EP400, score = 0.900). 

**Figure 16:**
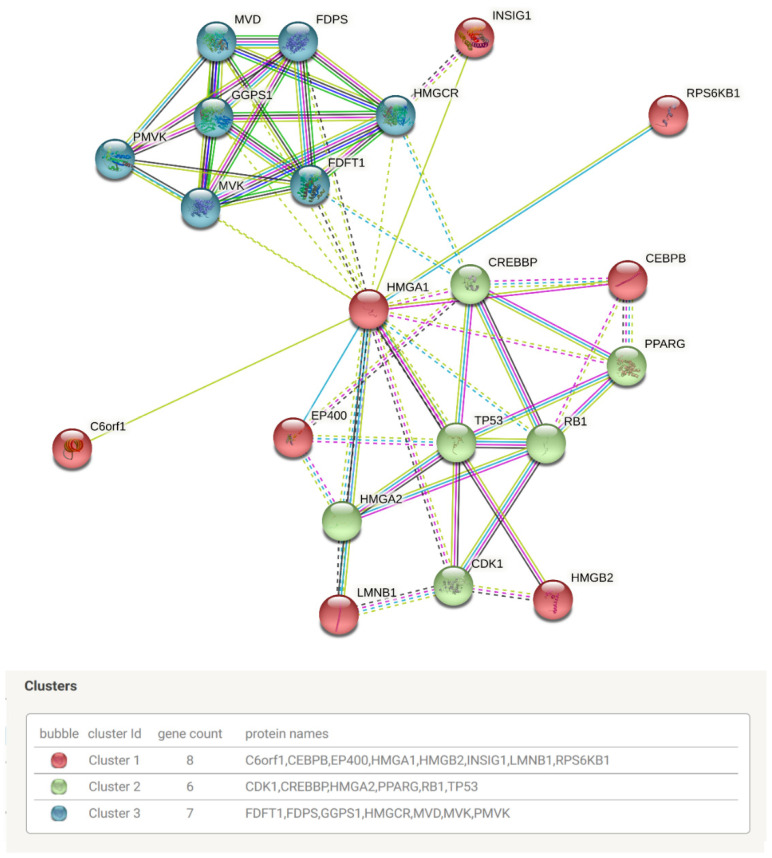
Clustered protein-protein interaction network of the HMGA1. Edges of clusters were indicated by dotted lines.

Clustering of the generated PPI network revealed three clusters of interacting proteins (Figure 16). Functional enrichment analysis against gene ontology found the network to be mostly enriched in cellular senescence and cell ageing, cell cycle control, transcription regulation, chromatin assembly and remodeling, cholesterol and isoprene biosynthesis for biological processes, and significantly enriched in chromatin and nucleoplasm for cellular components.

TCGA and TGEx data provided by the GEPIA platform was used to analyze the correlation between *HMGA1* expression level and those of genes coding revealed protein interactors in lung adenocarcinoma, lung squamous cell carcinoma, and normal lung cancer tissue (Table 3). The strongest positive correlation was found for *CDK1* (R_S_ = 0.61) and *LMNB1* (R_S_ = 0.54) in lung adenocarcinoma. The expression levels of *EP400*, *HMGB2*, *CDK1, PPARG,* and *MVK* were found to be significantly correlated with *HMGA1* expression level in both analyzed cancer subtypes but not in non-cancerous tissue of the lung. Expression levels of the *GGPS1* and *PMVK* genes were only significantly correlated with *HMGA1* expression in lung adenocarcinoma.

## 3. Discussion

In 1983, Lund et al. [17] discovered HMGA1 expression in cervical cancer HeLa S3 cells. Since then, an elevated expression of the protein or *HMGA1* gene has been confirmed in many cancers of epithelial and mesenchymal origin, e.g., breast cancer [18], gastric cancer [19], lung cancer [20], or glioblastoma [21]. The present study used big data to screen for *HMGA1* expression levels in various types of cancers. Its findings largely support the previously published observation that *HMGA1* overexpression is a common feature of cancers, even those with distinct tissue origins: tumors of the head and neck, breast, gastrointestinal, and reproductive system, as well as melanoma and lymphomas, demonstrated elevated *HMGA1* expression in comparison to non-cancerous tissue, followed by an increase in HMGA1 protein. Surprisingly, a few exceptions were also noted; for example, in kidney chromophobe and renal clear cell carcinoma, the *HMGA1* was underexpressed. This suggests the presence of distinct gene expression regulation mechanisms in these types of cancer. Indeed, tissue-specific upstream and downstream regulation of *HMGA1* was described in head and neck, thoracic, reproductive system cancer, and other abdominal tumors [8]. Taken together, the deregulation of *HMGA1* expression appears to be a hallmark of cancer and implies that *HMGA1* plays an essential role in carcinogenesis. but the mechanisms in which the expression is changed during carcinogenesis remain unclear. 

Earlier research showed higher expression of the *HMGA1* gene in lung tumors than in the normal lung tissue [10,11,12]. Our present reanalysis using currently available data by Oncomine and TNMplot found that *HMGA1* is overexpressed in lung cancer in comparison to unpaired normal lung tissue and to the non-cancerous tissue adjacent to the tumor. The data published so far cannot conclusively indicate that *HMGA1* expression differs between different histological subtypes of lung cancer. Upregulation of *HMGA1* gene expression has been reported in both adenocarcinoma and squamous cell carcinoma of NSCLC [20], and HMGA1 protein was detected in a high proportion of lung cancer tumors irrespective of the histological type [13]. No major differences in *HMGA1* gene or protein expression were reported between different NSCLC subtypes by Zhang et al. [14] and Zhang et al. [15]. However. Lin and Peng [16] detected higher levels of HMGA1 protein in the squamous cell carcinoma subtype compared to adenocarcinoma. Our present analysis of the various large datasets provided by Oncomine showed that *HMGA1* upregulation in a common phenomenon, concerning not only adenocarcinoma and squamous cell carcinoma but also large and small cell carcinomas of the lung. In addition, the *HMGA1* expression level was positively correlated with HMGA1 protein abundance in both lung adenocarcinoma and lung squamous cell carcinoma. Most likely, re-expression of the *HMAG1* gene occurs at the early stages of carcinogenesis in the lung and increases as the process progresses. HMGA1 protein has been found to be present in increased amounts in metaplasia, dysplasia, and carcinoma in situ of the bronchial epithelium [13]. These findings raise the question of whether *HMGA1* could be useful in distinguishing between normal and neoplastic lung tissue. For example, early research demonstrated that *HMGA1* expression could be useful in differentiating between thyroid follicular carcinoma and adenoma [22]. Our TMNplot analysis indicates that the *HMGA1* expression level could discriminate with good sensitivity and specificity between cancerous and adjacent non-cancerous tissues in both lung adenocarcinoma and squamous cell carcinoma. Thus, it could be concluded that the *HMGA1* expression level could be a lung cancer biomarker.

With *HMGA1* overexpression being a common feature of lung cancer, the study then examined the molecular mechanisms responsible for its upregulation. Further research showed that growth factors, cancer-associated mutations (KRAS, mutated APC), or cancer-related transcription factors (cMYC) could up-regulate *HMGA1* in specific contexts [23]. Little is known about the role of *HMGA1* mutations in determining the function and expression level of the *HMGA1* in cancer. In invasive triple-negative breast cancer cells, mutating the three HMGA1 serines (99, 102, and 103) phosphorylated by casein kinase 2 (CK2) results in decreased HMGA1 secretion and disrupts the invasive properties of the cell line [24]. Mutation of serine 102 of *HMGA1*, a CK2 phosphorylation site, was found to restore the efficacy of gefitinib through the reactivation of the downstream signaling pathway of EGFR in drug-resistant NSCLC cells [25]. 

Our own analysis of the data provided by cBioPortal found that the genomic alterations of the *HMGA1* gene occur very rarely in lung cancer tissue. Collective frequency of gene amplification, gene deletion, and point mutation slightly exceeded 1% of 7276 analyzed lung cancer cases and the detected alterations were restricted to adenocarcinoma and squamous cell carcinoma subtypes. This is in agreement with Klett et al. [26], who showed that *HMGA1* was among the top 5% of more than 19,000 genes, with the lowest mutation frequency analyzed in more than 9000 cancerous samples from TCGA. As could be expected, a positive correlation was found between *HMGA1* expression level and the copy number of the gene. However, the low occurrence of *HMGA1* amplification or point mutation in lung cancer excludes the possibility that genomic alterations of *HMAG1* are responsible for the common overexpression of *HMGA1* in this type of tumor. In contrast, a negative correlation was found between the mRNA expression level and the DNA methylation profile of *HMGA1* in lung cancer. 

TCGA data provided by the UALCAN platform indicated that the *HMGA1* promoter methylation level was significantly decreased in both lung adenocarcinoma and lung squamous cell carcinoma tissue in comparison with normal lung tissue. Another study based on TCGA data by Klett et al. [26] demonstrated that hypomethylation of 9 from 21 analyzed CpGs in *HMGA1* was an indicator of increased gene expression in breast cancer, lung adenocarcinoma, and thyroid cancer. *HMGA*, as one of the pluripotency-related genes, acquired 5-methylcytosines in their promoters, which were repressed during differentiation of human embryonic stem (hES) cells into ventral midbrain-type neural precursor cells and then into dopamine neurons. Promoter methylation was preserved to maintain a silencing state [27]. It also was shown that *HMGA1* was suppressed in hepatocellular carcinoma cells by human menstrual-blood-derived stem cells via amending 5-hydroxymethylcytosine and 5-methylcytosine abundance at its regulatory region [28]. Considering the above. the *HMGA1* gene may be a potential candidate for DNA methylation targeting anti-cancer therapy. 

Our findings indicate some connection between *HMGA1* expression and the sex and smoking history of lung cancer patients. While Lin and Peng [16] report that tissue HMGA1 expression was more frequently observed in men than women, this was contradicted by Zhang et al. [14] and Zhang et al. [15]. In our study, the *HMGA1* expression level was consistently higher among men in both lung adenocarcinoma and lung squamous cell carcinoma. The gender-specific biological and clinical features of lung cancer have been described previously [29,30], suggesting that lung cancer in women and men are distinct entities, mainly because of the involvement of genetic alterations and hormonal factors. Rouquetta et al. [3] observed a higher rate of *EGFR* mutations in women. ER and EGFR were also expressed more frequently in women and there was a positive link between the expression of EGFR and of ER. As *HMGA1* is an upstream negative regulator of the EGFR signaling pathway [25], it could be speculated that gender-specific *HMGA1* expression influences lung cancer cell behavior.

Among NSCLC patients, ever-smokers demonstrate higher overall mortality than never-smokers, and current smoking is an independent risk factor for a poorer prognosis [31]. Our present data suggests some differences in *HMGA1* expression levels related to smoking status. Generally, the level of *HMGA1* expression tends to be the lowest in non-smokers, of intermediate value in reformed smokers, and the highest in current smokers in both lung adenocarcinoma and squamous cell carcinoma. However, in the lung adenocarcinoma group, statistically significant differences were noted between the non-smoker group and both reformed smokers >15 years and current smokers. Jung et al. [32] compared TCGA data for the smoker’s lung adenocarcinoma and normal lung tissue; the analysis found that *HMGA1* is one of six genes up-regulated in LUAD, and simultaneously inversely correlated with DNA methylation level. Borderline differences in CpG methylation of *HMGA1* have been noted between smoking and never-smoking lung adenocarcinoma patients based on validation of clinical samples. Since cigarette smoke affects DNA methylation and thus is a critical factor in the development of lung cancer [33,34], it may, at least partially, account for the observed differences in *HMGA1* expression levels associated with smoking habits.

The significant role played by *HMGA1* expression in promoting growth, invasiveness, and migration of lung cancer cells in vitro has been confirmed [11,35]. Similarly, in the present study, *HMGA1* expression level was found to increase from non-tumor lung tissue through lung primary tumor to lung cancer metastasis. Zhang et al. [14] and Zhang et al. [15] report that high HMGA1 protein expression in NSCLC tissue was associated with tumor size, the presence of lymph node and distant metastases, and with higher advancement according to the TNM classification. Although differences were noted in the expression level between patients of different nodal statuses and stages of the disease in the present study, the observed differences were restricted to one histological subtype of lung cancer or to particular states of the analyzed factor. A connection was observed between *HMGA1* expression level and nodal status in lung squamous cell carcinoma but not in lung adenocarcinoma, and the only significant difference in level was observed between N0 and N1 cases. An increase in the median *HMGA1* expression level was stated between stage I and stages II and III in adenocarcinoma, and between stages I and II in squamous cell carcinoma. Contrary to expectations, no further increase in the median level was detected in the most advanced IV stage, probably because of the relatively low number of cases in this stage. 

Zhang et al. [14] found high HMGA1 protein expression to be associated with a shorter survival time and was stated as an independent negative prognostic factor in NSCLC patients. No such correlation was observed by Lin and Peng [16]. In the present study, survival analysis indicates the high *HMGA1* expression level is connected with poor survival of lung cancer patients. Overall and first progression survival of lung cancer patients was shorter when *HMGA1* expression was high, according to the Kaplan–Meier plotter. However, survival analysis in the context of clinicopathological factors suggests that the association between *HMGA1* expression and survival rate may be limited to specific subgroups of patients. For example, a high expression level of the *HMGA1* gene was connected to an increased risk of death in stage I patients but not in stage I or II. Similarly, the mentioned connection was detected in the adenocarcinoma subtype but not in squamous cell carcinoma of the lung. This is in line with the early findings of Sarhadi et al. [13], who report a correlation between the nuclear expression of the HMGA1 protein and shorter survival time among patients with lung adenocarcinoma but not squamous cell carcinoma. It is most likely that the effect of the *HMGA1* on the progression of lung cancer is dependent on some other factors. For instance, Zhang et al. [15] stated the increased amount of HMGA1 protein found in NSCLC tissue was connected with shorter overall survival compared to patients with a low amount of HMGA1. However, the amount of HMGA1 protein in cancer tissue was correlated with the amount of FOXM1 and G6PD. The proteins form a common pathway of transcriptional regulation with HMGA1 and were similarly associated with TNM stage and overall survival. 

To identify molecular contributors of HMGA1 that could mediate or assist its function during carcinogenesis in lung tissue, a functional network was created. HMGA1 was found to cooperate with proteins that can be grouped into three functional clusters. The first cluster was mostly enriched by protein involved in transcriptional regulation and chromatin assembly and remodeling (*C6orf1*, *CEBPB*, *EP400*, *HMGB2*, *LMNB1*, and *RPS6KB1*). For most of these proteins, there is some evidence that they are concerned with neoplastic transformation in the lung. Ribosomal proteins S6 kinase B1 (RPS6KB1, p70S6K) are critical components of mTOR signaling, that have been found to increase the growth of NSCLC cells in vivo and inhibit tumor metastasis through repression of epithelial-mesenchymal transition [36]. The AMPK-CEBPB-PDL1 signaling pathway enhances the proliferation of NSCLC cells [37]. Lamin B1 is overexpressed in lung adenocarcinoma cells and promotes the proliferation of lung cancer cells via the AKT pathway [38]. p400 is an important mediator of downregulation of EGFR and apoptosis induced by E1A protein of human adenovirus [39]. By sharing a functional connection with the mentioned protein, HMGA1 could be involved in the most important signaling pathways driving lung carcinogenesis. Additionally, we found the expression level of *HMGA1* to be positively correlated with the expression of *HMGB2* and *EP400* genes in both lung adenocarcinoma and lung squamous cell carcinoma but not in normal lung tissue, which could suggest that co-expression of these genes may be crucial for malignant transformation. 

Functional enrichment analysis indicated that the second cluster within the built PPI network is an assembly of proteins involved in cellular senescence and cell cycle control (TP53, RB1, CDK1, CREBBP, PPARG, and HMGA2). Previously, bioinformatics analyses conducted by Zhou et al. [40] found *TP53* and *HMGA1* to be two of the main 10 genes belonging to the miRNA-gene interaction network, and may be crucial in the pathogenesis of NSCLC. However, the proteins are also believed to be engaged in other cellular processes essential for lung neoplastic transformation, such as cell adhesion, invasion and migration. For example, CDK1 positively regulated the stemness of lung cancer cells [41] and mediated the signaling for proliferation, invasion, and migration in NSCLC cells [42]. Loss of *CREBBP* drives tumorigenesis in SCLC by reducing histone acetylation and transcription of cellular adhesion genes [43]. Activation of *PPARG* expression may inhibit the progression of LSCC through the regulation of LSCC upstream regulators and downstream marker genes involved in tumor cell proliferation and protein polyubiquitination/ubiquitination [44]. Additionally, CREB binding protein (CREBBP) directly influences the HMGA1 function. Munshi et al. [45] demonstrated CREBBP acetylate HMGI(Y) modulating IFN-beta transcriptional regulation. Our present findings indicate that the *HMGA1* gene expression level correlated with expression of both *CDK1* and *PPARG* in lung adenocarcinoma and squamous cell carcinoma but not in normal lung tissue; this supports the findings that the interaction the HMGA1 with these CDK1 and PPARG are important for lung carcinogenesis. 

Proteins involved in cholesterol, mevalonate, and isoprene biosynthesis (FDFT1, FDPS, GGPS1, HMGCR, MVD, MVK, PMVK, and INSIG1) were revealed to be *HMGA1* interactors. An association between HMGA1 and adipogenesis has been described earlier. HMGA1 has been found to play an essential role in adipocytic cell growth and differentiation in murine 3T3-L1 adipocytes [46]. Reduced fat mass and impaired adipogenesis were demonstrated in transgenic mice overexpressing *HMGA1* in both white and brown adipose tissues compared to wild-type mice [47]. In humans, the *HMGA1* IVS5-13insC variant (rs146052672), which resulted in a decrease in mRNA and protein levels of HMGA1, was significantly associated with metabolic syndrome and negatively correlated with serum HDL-cholesterol [48,49]. The variant was also connected with the elevated risk of acute myocardial infarction independently of diabetes mellitus type 2 and other classic cardiovascular risk factors like gender, hypertension, and obesity [50]. There is some evidence that the relationship between the *HMGA1* and cholesterol biosynthesis may be significant in neoplastic transformation. Our findings indicate that the *MVK*, *GGPS1* and *PMVK* expression was correlated with *HMGA1* gene expression in lung cancer tissues, but not in normal lung tissue. Previously, Treff et al. [51] demonstrated that cholesterol biosynthesis genes are decreased in human MCF-7 mammary adenocarcinoma cells with *HMGA1a*-induced overexpression. Simultaneously, total cholesterol was significantly depleted and sensitivity to epidermal growth factor activation of ERK phosphorylation was significantly elevated in cells overexpressing *HMGA1a*. 

Taken together, the findings from the bioinformatical analysis indicate that elevated *HMGA1* expression is a common feature of many malignant tumors, including lung cancer. *HMGA1* overexpression in lung cancer could result from reduced *HMGA1* methylation and this is connected with some clinicopathological features and adverse prognosis of lung cancer patients. HMGA1 could be an essential element of lung carcinogenesis through the interaction with proteins involved in e.g., cell cycle control, transcription regulation, and cholesterol and isoprene biosynthesis. Considering the carcinogenic properties of *HMGA1* and its connection with poor prognosis in many types of cancer, some approaches were previously made to invent an in vitro diagnostic kit to quantitatively determine the amount of HMGA1b in peripheral blood of cancer patients [52]. In light of the results of our analysis, the evaluation of *HMGA1* expression level may be of great value in the management of lung cancer patients. Especially, the possibility to identify very little amounts of *HMGA1* transcripts directly in the blood specimens could either allow for early diagnosis or the monitoring of the efficacy of lung cancer therapy. Moreover, HMGA1 protein-targeted therapies based on aptamers or antisense RNA, as well as *HMGA1* gene inhibitors, were developed to slow down the proliferation of cancer cells or to increase their sensitivity to chemotherapy [9]. High expression levels of *HMGA1* mRNA in lung tumors and relatively low expression levels in non-cancerous tissue of the lung allow us to assume that such therapies would have clinical advantages in lung cancer because of presumed low toxicity toward healthy cells. 

## 4. Materials and Methods

### 4.1. Gene Expression Analysis

#### 4.1.1. Oncomine

The Oncomine database [53] (https://www.oncomine.org, accessed on 1 January 2022) was used: (1) to analyze the *HMGA1* mRNA expression level in a variety of human cancers, where threshold settings were as follows: gene ranking of the top 10%, change ≥ 2, *p*-value ≤ 1 × 10^−4^, and (2) to screen *HMGA1* mRNA expression level in different histological subtypes of lung cancer. All statistical methods and statistical values were obtained directly from the mentioned database. 

#### 4.1.2. TIMER2.0

The Gene_DE module of the Tumor Immune Estimation Resource 2.0 [54] (http://timer.cistrome.org, accessed on 1 January 2022) was used to study the differential expression between tumor and adjacent normal tissues for *HMGA1* gene across TCGA tumors. The distributions of gene expression levels are displayed using box plots. The statistical significance (*p*-value) was computed by the Wilcoxon test.

#### 4.1.3. TMNplot

The TMNplot web tool [55] (https://tnmplot.com, accessed on 1 January 2022) was used: (1) to display pan-cancer changes in *HMGA1* expression based on RNA-seq data from TCGA, genotype-tissue expression (GTEX), therapeutically applicable research to generate effective treatment (TARGET); significant differences are typed red and marked with an asterisk, (2) to compare *HMGA1* expression level in lung cancer and non-tumor lung tissues based on RNA-seq and DNA chip data. The normal and tumor samples were compared by the Mann–Whitney U-test, matched tissues with adjacent samples were compared using the Wilcoxon test. Normal, tumorous, and metastatic tissue gene comparison was analyzed using the Kruskal–Wallis test and Dunn’s test. 

### 4.2. Prognosis and Survival Analysis

#### 4.2.1. Ualcan

TCGA data available on the UALCAN portal [56] (http://ualcan.path.uab.edu/index.html, accessed on 1 January 2022) was used to validate the association between the *HMGA1* expression level with selected clinical features in the lung adenocarcinoma and lung squamous carcinoma. The results were presented in box-whisker plots with minimum, q1, median, q3, and maximum values. The significance of the difference was estimated by Student’s t-test considering unequal variance.

#### 4.2.2. Kaplan–Meier Plotter

The Kaplan–Meier plotter [57] (http://kmplot.com/analysis/index.php?p=service, accessed on 1 January 2022) was applied to evaluate the prognostic value of *HMGA1* expression level for overall survival and first progression survival in lung cancer, lung adenocarcinoma, and lung squamous cell carcinoma. Kaplan–Meier plots were drawn using “HMGA1” as an input query, array quality control was set as “exclude biased arrays”; patients were split by median; the desired Affy ID 206074_s_at was valid.

#### 4.2.3. PrognoScan

The PrognoScan [58] (http://dna00.bio.kyutech.ac.jp/PrognoScan/index.html, accessed on 1 January 2022) was used to evaluate the connection between *HMGA1* expression level and overall and relapse-free survival in lung adenocarcinoma and lung squamous cell carcinoma. Cox *p*-values and hazard ratio with a 95% confidence interval were calculated according to HMGA1 mRNA level (high vs. low).

### 4.3. DNA Alteration and Methylation of the HMGA1 Gene 

#### 4.3.1. cBioPortal 

The genomic characteristics of *HMGA1* in lung cancers were analyzed using the cBioPortal for Cancer Genomics [59] (v3.7.28; http://www.cbioportal.org, accessed on 1 January 2022). Combined querying of 7276 patients/7799 samples from 27 studies was performed. The incidence of different alterations of the studied gene was assessed in lung cancer cases and, specifically, in NSCLC histological types. Additionally, the association between *HMGA1* mRNA expression level and copy number alteration, and between the mRNA level of the gene and methylation beta-value (HM450, 492 cases), was analyzed. Spearman’s and Pearson’s correlation coefficients were calculated.

#### 4.3.2. Oncomine 

The Oncomine database [53] (https://www.oncomine.org, accessed on 1 January 2022) was used to extract and analyze data about the copy-number alteration of the *HMGA1* in different histological subtypes of lung cancer. 

#### 4.3.3. UALCAN 

TCGA data available on the UALCAN portal [56] (http://ualcan.path.uab.edu/index.html, accessed on 1 January 2022) was used to compare the promoter methylation level in the lung adenocarcinoma and lung squamous carcinoma and healthy lung tissue. The boxplot represents beta values of CpG probes located up to 1500 bp upstream of the gene’s start site. The beta value is the ratio of the methylated probe intensity to the sum of methylated and unmethylated probe intensity. The beta value ranges from 0 to 1. 

### 4.4. Protein Analysis

#### cBioPortal

Association between mRNA expression level vs. protein amount was assessed with the cBioPortal for Cancer Genomics [59] (v3.7.28; http://www.cbioportal.org, accessed on 1 January 2022). Separate analysis for lung adenocarcinoma (querying 4159 patients/4309 samples from 12 studies) and lung squamous cell carcinoma (querying 1256 patients/samples from 4 studies) were conducted. Spearman’s and Pearson’s correlation coefficients were calculated.

### 4.5. Interaction Network Analysis

#### 4.5.1. STRING 

The STRING database [60] (https://string-db.org/, accessed on 1 January 2022) was used to build a protein–protein interaction (PPI) network querying the protein “HMGA1” and organism “Homo sapiens”. The main parameters were set as follows: the minimum required interaction score was 0.7 and no more than 50 interactors to show. K-means clustering of the generated PPI network was performed with a pre-set of three clusters. 

#### 4.5.2. GEPIA

Correlation between *HMGA1* expression level and expression level of the selected genes was evaluated in TCGA and TGEx data provided by gene expression profiling interactive analysis (GEPIA) [61] (http://gepia.cancer-pku.cn/index.html, accessed on 1 January 2022). Spearman’s correlation coefficient and *p*-values were calculated for each correlation. 

### 4.6. Statistical Analysis

Computing the appropriate statistics was provided by data platforms utilized in the present study. In the Oncomine database, Student’s t-test was used to analyze the differential expression. The statistical significance was computed in the TIMER database by the Wilcoxon test. In the TNM plot database, to assess the significance of the expression level differences, the Mann–Whitney U test was used when two subgroups of samples were compared. Meanwhile, the Kruskal–Wallis test, followed by the Dunn post hoc test, was applied for analyzing the two-class differential expression analysis. When differences in expression levels with respect to clinical parameters were analyzed by the UALCAN, t-test assuming the unequal variance was calculated. Spearman and/or Pearson correlation coefficients were estimated for analysis of gene–gene expression level by the GEPIA, gene-to-protein expression level, gene expression-to-copy number value, and gene expression-to-methylation profile by the cBioPortal. To compare survival curves, the log rank test and hazard ratio (HR) with a 95% confidence interval (CI) were calculated. A univariate Cox regression model was applied to calculate the HR and Cox *p* value in the PrognoScan. In all above-mentioned analyses *p* < 0.05 was assumed to be significant. 

## Figures and Tables

**Table 1:** Survival analysis of *HMGA1* expression regarding selected clinicopathological factors in lung cancer by Kaplan–Meier plotter.

Variable	Overall Survival			First Progression Survival		
	N	Hazard Ratio	*p*-Value	N	Hazard Ratio	*p*-Value
Non-restricted analysis	1925	1.78 (1.57–2.03)	**<1 × 10^−16^**	982	1.60 (1.32–1.94)	**1.7 × 10^−6^**
Grade						
I	201	1.34 (0.93–1.92)	0.1101	140	1.21 (0.78–1.87)	0.4016
II	310	1.70 (1.24–2.34)	**0.0009**	165	1.33 (0.88–2.02)	0.1729
III	77	1.59 (0.82–3.07)	0.1676	51	0.68 (0.30–1.54)	0.3572
Histology						
adenocarcinoma	719	1.93 (1.52–2.45)	**4.7 × 10^−8^**	461	1.79 (1.30–2.46)	**0.0003**
squamous cell carcinoma	524	1.15 (0.91–1.46)	0.2403	141	1.24 (0.74–2.07)	0.4168
Stage						
I	577	3.01 (2.24–4.05)	**2.3 × 10^−14^**	325	1.48 (0.95–2.29)	**0.0810**
II	244	1.33 (0.92–1.92)	0.1239	130	0.91 (0.54–1.52)	0.7062
III	70	1.00 (0.57–1.74)	0.9962	19	NA	NA
IV	4	NA	NA	0	NA	NA
AJCC stage T						
1	437	1.66 (1.24–2.22)	**0.0005**	177	1.75 (1.04–2.93)	**0.0318**
2	589	1.33 (1.07–1.67)	**0.0108**	351	1.19 (0.88–1.60)	0.2636
3	81	1.15 (0.70–1.89)	0.5938	21	1.89 (0.71–5.07)	0.1968
4	46	1.47 (0.78–2.77)	0.2355	7	NA	NA
AJCC stage N						
0	781	1.39 (1.12–1.71)	**0.0023**	374	1.31 (0.95–1.81)	0.1046
1	252	1.38 (1.01–1.88)	**0.0455**	130	1.53 (0.97–2.42)	**0.0640**
2	111	1.23 (0.82–1.84)	0.3076	51	2.47 (1.20–5.08)	**0.0110**
0	781	1.39 (1.12–1.71)	**0.0023**	374	1.31 (0.95–1.81)	0.1046
AJCC stage M						
0	681	1.46 (1.18–1.79)	**0.0004**	195	1.35 (0.82–2.25)	0.2375
1	10	NA	NA	0	NA	NA
Sex						
female	714	2.08 (1.63–2.64)	**1.1 × 10^−9^**	468	1.40 (1.05–1.86)	**0.0218**
male	1100	1.59 (1.36–1.87)	**7.6 × 10^−9^**	514	1.61 (1.24–2.09)	**0.0003**
Smoking history						
never-smokers	205	2.20 (1.22–3.69)	**0.0071**	193	1.73 (1.06–2.82)	**0.0255**
present or reformed smokers	820	1.71 (1.38–2.11)	**4.9 × 10^−7^**	603	1.53 (1.20–1.96)	**0.0006**

**Table 2:** *HMGA1* expression and survival data of lung cancer patients using the PrognoScan database.

Dataset	Subtype	End-Point *	Probe ID	N	Cut-Point	Cox *p*-Value	HR [95% CI]
jacob-00182-CANDF	LUAD	OS	210457_x_at	82	0.32	0.1758	1.25 [0.90–1.73]
jacob-00182-CANDF	LUAD	OS	206074_s_at	82	0.48	**0.0547**	1.99 [0.99–4.03]
HARVARD-LC	LUAD	OS	39704_s_at	84	0.88	**0.0073**	1.67 [1.15–2.42]
jacob-00182-HLM	LUAD	OS	210457_x_at	79	0.80	0.5391	0.89 [0.60–1.30]
jacob-00182-HLM	LUAD	OS	206074_s_at	79	0.23	0.9811	1.00 [0.67–1.51]
MICHIGAN-LC	LUAD	OS	L17131_rna1_at	86	0.20	**0.0998**	1.48 [0.93–2.35]
jacob-00182-MSK	LUAD	OS	206074_s_at	104	0.81	**0.0235**	1.83 [1.08–3.08]
jacob-00182-MSK	LUAD	OS	210457_x_at	104	0.83	**0.0138**	1.62 [1.10–2.37]
GSE13213	LUAD	OS	A_23_P42331	117	0.11	**0.0606**	0.34 [0.11–1.05]
GSE13213	LUAD	OS	A_24_P222043	117	0.81	**0.0151**	1.35 [1.06–1.73]
GSE31210	LUAD	OS	210457_x_at	204	0.68	0.1824	0.74 [0.48–1.15]
GSE31210	LUAD	OS	206074_s_at	204	0.61	**0.0056**	2.01 [1.23–3.29]
jacob-00182-UM	LUAD	OS	210457_x_at	178	0.60	0.6309	1.06 [0.83–1.35]
jacob-00182-UM	LUAD	OS	206074_s_at	178	0.66	**0.0627**	1.39 [0.98–1.96]
GSE31210	LUAD	RFS	210457_x_at	204	0.35	0.5440	0.90 [0.63–1.27]
GSE31210	LUAD	RFS	206074_s_at	204	0.78	**0.0000**	2.34 [1.62–3.37]
GSE4573	LUSC	OS	206074_s_at	129	0.37	0.1601	1.45 [0.86–2.45]
GSE4573	LUSC	OS	210457_x_at	129	0.20	0.4697	1.13 [0.81–1.59]
GSE17710	LUSC	OS	32619	56	0.84	0.8742	1.06 [0.53–2.09]
GSE17710	LUSC	OS	29455	56	0.73	0.6085	0.80 [0.35–1.85]
GSE17710	LUSC	OS	39302	56	0.55	0.6607	1.10 [0.72–1.66]
GSE17710	LUSC	RFS	39302	56	0.55	0.6389	1.10 [0.74–1.64]
GSE17710	LUSC	RFS	32619	56	0.79	0.8784	0.95 [0.48–1.89]
GSE17710	LUSC	RFS	29455	56	0.11	0.6048	0.82 [0.38–1.77]

* OS—overall survival, DSS—disease-specific survival, RFS—relapse-free survival.

**Table 3:** Correlation between expression levels of the *HMGA1* and the genes coding putative interacting proteins in lung adenocarcinoma, lung squamous cell carcinoma, and normal lung tissue. Calculated by GEPIA.

	Lung Adenocarcinoma		Lung Squamous Cell Carcinoma		Normal Lung Tissue	
	R Spearman	*p*-Value	R Spearman	*p*-Value	R Spearman	*p*-Value
*C6orf1*	0.02	0.72	0.21	**3.30 × 10^−6^**	0.21	**0.00027**
*CEBPB*	0.17	**1.8 × 10^−4^**	−0.21	**2.4 × 10^−6^**	0.27	**3.9 × 10^−6^**
*EP400*	0.21	**2.2 × 10^−6^**	0.26	**3.10 × 10^−9^**	−0.09	0.13
*HMGB2*	0.39	**1.10 × 10^−18^**	0.25	**4.20 × 10^−8^**	−0.06	0.33
*LMNB1*	0.54	**8.70 × 10^−38^**	0.38	**6.10 × 10^−18^**	0.43	**2.10 × 10^−14^**
*RPS6KB1*	0.31	**3.90 × 10^−12^**	0.26	**8.90 × 10^−9^**	0.10	**0.081**
						
*CDK1*	0.61	**3.00 × 10^−50^**	0.27	**2.80 × 10^−9^**	0.06	0.35
*CREBBP*	0.08	0.073	0.18	**5.00 × 10^−5^**	−0.27	**3.30 × 10^−6^**
*HMGA2*	0.31	**2.00 × 10^−12^**	0.24	**8.70 × 10^−8^**	0.18	**0.002**
*PPARG*	0.20	**6.00 × 10^−6^**	0.15	**8.00 × 10^−4^**	−0.02	0.062
*RB1*	0.03	0.54	−0.04	0.41	−0.07	0.25
*TP53*	0.01	0.77	0.18	**6.90 × 10^−4^**	0.08	0.19
						
*INSIG1*	0.12	**9.40 × 10^−3^**	0.13	**5.20 × 10^−3^**	0.29	**8.2 × 10^−7^**
*FDFT1*	0.01	0.81	0.22	**5.60 × 10^−7^**	0.28	**1.40 × 10^−6^**
*FDPS*	0.30	**1.30 × 10^−11^**	0.25	**2.90 × 10^−8^**	0.2	**5.00 × 10^−4^**
*GGPS1*	0.10	**0.029**	0.04	0.44	−0.01	0.9
*HMGCR*	0.23	**2.00 × 10^−7^**	0.22	**1.50 × 10^−6^**	0.25	**2.10 × 10^−5^**
*MVD*	0.19	**2.60 × 10^−5^**	0.18	**7.20 × 10^−5^**	0.37	**5.00 × 10^−11^**
*MVK*	0.12	**0.0083**	0.19	**1.50 × 10^−5^**	−0.01	0.88
*PMVK*	−0.09	**0.042**	0.00	0.98	−0.01	0.9

## Data Availability

Links to publicly archived datasets analyzed: https://www.oncomine.org, http://timer.cistrome.org, https://tnmplot.com, http://ualcan.path.uab.edu/index.html, http://kmplot.com/analysis/index.php?p=service, http://dna00.bio.kyutech.ac.jp/PrognoScan/index.html, http://www.cbioportal.org, https://string-db.org/, and http://gepia.cancer-pku.cn/index.html, accessed on 1 January 2022.
